# Effects of Mixed Cotton Stalk and Sugar Beet Pulp Microsilage on Growth Performance, Meat Quality, Muscle Metabolism, and Intestinal Microbiota in Suffolk Rams

**DOI:** 10.3390/ani16091378

**Published:** 2026-04-30

**Authors:** Nuerminamu Aihemaiti, Yongkuo Li, Tao Li, Linhai Song, Haoran Liu, Zhanpeng Wang, Wei Shao, Wanping Ren, Liang Yang

**Affiliations:** 1 College of Animal Science, Xinjiang Agricultural University, Urumqi 830052, China; 17590816304@163.com (N.A.); 13289929530@163.com (Y.L.); 18146428958@163.com (T.L.); 15009360416@163.com (L.S.); 13379301223@163.com (H.L.); 18196132009@189.com (Z.W.);; 2 Xinjiang Herbivore Nutrition Laboratory for Meat & Milk, Urumqi 830052, China

**Keywords:** Suffolk rams, cotton stalks, microsilage, meat production performance, metabolomics, gut microbiota

## Abstract

In modern intensive mutton sheep farming, the high cost and limited supply of conventional feed resources necessitate the exploration of sustainable alternatives. Cotton stalks and sugar beet pulp, abundant agricultural by-products in China, have potential as ruminant feed after proper fermentation treatment, yet their systematic application in sheep production remains underinvestigated. This study evaluated the effects of replacing whole-plant corn microsilage with mixed fermented feed (cotton stalks and sugar beet pulp, 1:1 dry matter ratio) on Suffolk rams (*n* = 84, 4 months old). Animals were randomly assigned to four groups: control (CK, 0% replacement), MS30 (30% replacement), MS60 (60% replacement), and MS90 (90% replacement). After a 15-day adaptation, the 120-day feeding trial assessed growth performance, slaughter characteristics, meat quality, muscle metabolomics (LC-MS), and jejunal microbiota (16S rRNA sequencing). The MS60 group significantly outperformed the CK group in final body weight, carcass weight, and net weight gain (*p* < 0.01), slaughter rate (*p* < 0.05), and meat tenderness (*p* < 0.05). Fatty acid composition was optimized, with lower SFAs (*p* < 0.01) and higher MUFAs (*p* < 0.01). Metabolomic analysis revealed 206 differentially abundant metabolites, with significant enrichment in linoleic acid metabolism, unsaturated fatty acid biosynthesis, and primary bile acid synthesis pathways. The MS60 group exhibited significantly altered jejunal microbiota structure (*p* < 0.05), including increased Patescibacteria abundance (*p* < 0.05) and decreased Bifidobacterium (*p* < 0.001). Replacing 60% of whole-plant corn microsilage with cotton stalk–beet pulp mixed microsilage effectively improved production performance, meat quality, and fatty acid profiles in Suffolk rams, while modulating muscle metabolism and intestinal microbiota structure. These findings provide a practical strategy for sustainable sheep farming utilizing regional agricultural by-products.

## Simple Summary

In this study, cotton stalk–beet pulp mixed microsilage was used to replace whole-plant corn silage, and its effects on Suffolk rams were investigated. Eighty-four 4-month-old sheep were randomly divided into four groups, with 0%, 30%, 60%, and 90% replacement of whole-plant corn microsilage, respectively. They were fed for 120 days. The results showed that the 60% replacement (MS60) group exhibited superior growth performance, slaughter traits, meat tenderness, and a healthier fatty acid profile compared to other groups. Specifically, the MS60 group had significantly higher body weight and carcass weight, lower shear force (indicating more tender meat), decreased saturated fatty acids, and increased monounsaturated fatty acids. The study demonstrated that this substitution method could effectively improve the quality of mutton and provide new ideas for green farming.

## 1. Introduction

In modern intensive mutton sheep farming, enhancing meat production efficiency and optimizing mutton quality are the core objectives for driving the growth of industrial economic benefits. Suffolk sheep, as a world-renowned meat breed, hold a significant position in international mutton sheep production and crossbreeding improvement due to their rapid growth rate and excellent meat production performance [1,2]. However, the traditional farming model faces challenges such as high farming costs, a single feed source, and insufficient supply of high-quality forage. The development and utilization of unconventional feed resources have thus become a crucial direction for the sustainable development of the livestock industry. China’s cotton and sugar industries generate substantial amounts of cotton stalk and beet pulp by-products annually. Cotton stalks are rich in cellulose, and beet pulp has a high sugar content, making both potential feed resources for ruminants [3]. Nevertheless, the high lignin content and poor palatability of untreated cotton stalks, along with the high moisture content and difficulty in storage of beet pulp, limit their application as feed. Microsilage technology, which involves adding microbial inoculants and fermenting under anaerobic conditions, can degrade crude fiber, produce organic acids, and effectively improve the nutritional value and palatability of feed [4]. Mixing cotton stalks with beet pulp for microsilage is expected to yield a high-quality roughage source through nutritional complementarity and fermentation synergism.

Diet composition is a key factor influencing the growth performance, carcass composition, and meat quality of ruminants [5,6]. Studies have shown that fermented feed can alter the fatty acid composition of mutton, increasing the proportion of polyunsaturated fatty acids [7]. The formation of meat quality is closely related to metabolic processes such as post-mortem muscle glycolysis, fat oxidation, and protein degradation [8]. As a central hub in the body’s metabolic regulation, intestinal microbiota play a crucial role in fiber degradation, short-chain fatty acid synthesis, and immune regulation. Changes in the source and content of dietary fiber can reshape the intestinal microbial community, thereby affecting energy utilization efficiency and host health [9]. Currently, systematic reports on the application of mixed microsilage feed made from cotton stalks and beet pulp in mutton sheep production, especially regarding its impact on meat quality, muscle metabolic profiles, and the interaction mechanisms with intestinal microbiota, are scarce. We hypothesized that replacing whole-plant corn microsilage with different proportions (30%, 60%, and 90%) of cotton stalk and beet pulp mixed microsilage would affect growth performance in Suffolk rams, with the optimal level to be determined experimentally, and that this optimal level would improve meat quality, associated with altered muscle metabolic pathways and a reshaped jejunal microbial community. Therefore, this study used Suffolk rams as experimental subjects to systematically evaluate the effects of different proportions of cotton stalk and beet pulp mixed microsilage on growth performance, slaughter performance, meat quality, muscle fatty acid composition, muscle metabolomic characteristics, and intestinal microbiota, with the objective of exploring associated changes in muscle metabolomics and gut microbiota, thereby revealing potential interactions among diet, microbiota, and metabolism.

## 2. Materials and Methods

### 2.1. Experimental Details and Ethical Policy

This experiment was conducted at Xinjiang Xin’ao Animal Husbandry Co., Ltd., Manas County, Xinjiang, China, from May to December 2025. The entire experimental process strictly adhered to the national “Regulations for the Administration of Laboratory Animals” and the “Guidance Suggestions for the Humane Treatment of Laboratory Animals” issued by the Ministry of Science and Technology, ensuring standardized operations and the full protection of experimental animal welfare. The experimental protocol was reviewed and approved by the Experimental Animal Welfare Ethics Committee of Xinjiang Agricultural University prior to implementation (Approval Number: xjau-aw-2025-0217; Approval Date: 5 February 2025) ensuring that the study complied with ethical standards.

### 2.2. Experimental Design and Animal Management

A total of 84 healthy Suffolk rams, aged approximately 4 months with an average initial body weight of 32.36 ± 2.92 kg, were used in this study. The 84 rams were randomly assigned to the four treatment groups using a random number generator in Excel (Microsoft Corp., Redmond, WA, USA): control group (CK), MS30, MS60, and MS90. Each group consisted of 21 rams, divided into three replicates with seven rams per replicate housed in the same pen. The CK group was fed a basal diet containing 21% whole-plant corn microsilage. In the experimental diets, whole-plant corn microsilage in the basal diet was replaced with mixed cotton stalk–beet pulp microsilage at levels of 0% (CK), 30% (MS30), 60% (MS60), and 90% (MS90) on a dry matter basis (Table 1). All diets were formulated to be isoenergetic and isonitrogenous. The experiment included a 15-day adaptation period, followed by a 120-day formal experimental period.

### 2.3. Feeding Management

All experimental sheep were treated with uniform deworming and stomach strengthening before the start of the experiment. They were fed twice a day at 07:00 and 19:00, and allowed to eat and drink ad libitum. The basal TMR was formulated according to China’s “Feeding Standards for Meat Sheep” (NY/T 816-2021) [10]. The preparation process for the cotton stalk–beet pulp mixed microsilage used in the trial was as follows: Cotton stalks were shredded and crushed to approximately 1 cm in length. They were then mixed with fresh beet pulp (moisture content: (75 ± 3)%) at a dry matter ratio of 5:5. Then uniformly sprayed with a specialized cotton stalk fermentation compound inoculant (containing ≥ 1 × 10^8^ CFU/g total viable bacteria, accounting for 0.1% of fresh weight), and 0.2% urea and 0.05% salt were added. Moisture content was adjusted to 60–65%, compacted to a density of approximately 900 kg/m^3^, sealed with polyethylene film, and fermented anaerobically at 25–30 °C for 120 days before use.

### 2.4. Growth Performance Assessment

On the morning before feeding at the beginning of the experiment and on the 120th day, all the experimental sheep were weighed fasting (without food for 12 h). The weight measurement was conducted using a commercial electronic scale (model: XK3150, Kai Feng Company (Taichung, China); accuracy: ±0.05 kg). Net weight gain was calculated by subtracting the initial weight from the final weight. During the experiment, feeding was carried out at fixed times, the amount of feed provided was accurately recorded, and the remaining feed was collected and weighed the next morning. The daily feed intake was calculated per pen, and then divided by the number of animals in that pen to obtain the average daily feed intake. The calculation formula is

Average daily feed intake = (Total feed provided − Total remaining feed)/(Number of days of the experiment × Number of animals in the pen).

Based on the total feed intake and total weight gain throughout the entire experiment period, the feed-to-weight ratio was calculated. The calculation formula is Feed-to-weight ratio = Total feed intake/Total weight gain.

### 2.5. Slaughter Performance Assessment

To enable a detailed investigation of the treatment effects on meat production and omics profiles, and in adherence to the 3R principles, a subset of animals from the control group (CK) and the group exhibiting the optimal growth performance (MS60) was selected for slaughter sampling and subsequent analyses. This extreme-group comparison approach maximizes the potential to detect biologically relevant differences. Consequently, the MS30 and MS90 groups were not subjected to slaughter or any omics analyses, and all findings related to meat quality, fatty acids, metabolomics, and microbiota are presented only for the CK and MS60 groups. The conclusions drawn from these analyses therefore apply solely to this comparison. Before slaughter, all the experimental sheep were fasted for 24 h and deprived of water for 12 h. The sheep were uniformly transported to the slaughterhouse (transport time approximately 25 min) for slaughter. The weights of all internal organs, sheep skin, lower limbs, and carcasses were measured and recorded. Carcass weight (kg): the weight of the entire trunk (including kidneys and perirenal fat) after slaughtering, bleeding, skin removal, head removal, hoof removal, and internal organ removal.

Slaughter rate (%) = Carcass weight/Pre-slaughter live weight × 100.

Net meat rate (%) = Net meat weight/Pre-slaughter live weight × 100.

Eye muscle area: The longissimus dorsi muscle was cut at the 12th to 13th thoracic vertebrae, and the cross-sectional contour was traced using sulfur paper. The eye muscle area (cm^2^) was measured using the transparent grid overlay method.

Back fat thickness: Subcutaneous fat thickness was directly measured 5 cm from the midline at the 12th to 13th ribs using a vernier caliper (model: INSIZE 1058-150C, Shanghai, China). Results are expressed in millimeters (mm).

### 2.6. Sample Collection and Preservation

Samples were collected immediately after slaughter. Approximately 300 g of the longissimus dorsi muscle was collected, divided into individual vials, and rapidly frozen in liquid nitrogen for the determination of fatty acid composition and muscle metabolomics; another 500 g of the longissimus dorsi muscle was taken for routine meat quality analysis. At the same time, the contents of the jejunum were collected, divided into 3 vials of 2 mL each, and rapidly frozen in liquid nitrogen for intestinal microbiota analysis. The remaining tissue samples were frozen at −20 °C for later analysis.

### 2.7. Meat Quality Determination

Approximately 24 h post-mortem, multiple meat quality indicators were measured. First, a portable meat pH meter (HI981036, Hanna Instruments, Cluj Napoca, Romania) was used to determine the muscle pH values at 45 min post-mortem (pH45 min) and 24 h post-mortem (pH_24h_). Meanwhile, a portable colorimeter (P-F-10, Konica Minolta Sensing, Inc., Tokyo, Japan) was employed to measure the lightness (L*_24h_), redness (a*_24h_), and yellowness (b*_24h_) values of the meat samples. In addition, a digital muscle tenderness meter (C-LM3B, College of Engineering, Northeast Agricultural University, Harbin, China) was utilized to measure the muscle shear force to evaluate tenderness. Drip loss was measured using the hanging method described by Li et al. [11]. At 24 h post-mortem, approximately 30 g of muscle samples (with dimensions of approximately 3 cm × 2 cm × 1 cm) were taken from the longissimus dorsi muscle, weighed, and recorded as W1. The samples were suspended by thin threads inside an inflated plastic bag, ensuring that the meat samples did not come into contact with the bag walls, and then allowed to stand for 24 h in a 4 °C refrigerator. After removal, the surface moisture was gently blotted with filter paper, and the samples were weighed again and recorded as W2. The drip loss rate was calculated using the following formula: drip loss rate (%) = (W1 − W2)/W1 × 100. Additional muscle samples were taken for the determination of intramuscular protein content and intramuscular fat content. The protein content was measured in accordance with the “National Food Safety Standard—Determination of Protein in Foods” (GB 5009.5-2016) [12], and the fat content was measured in accordance with the “National Food Safety Standard—Determination of Fat in Foods” (GB 5009.6-2016) [13]. All samples for measurement were stored under corresponding conditions for future use.

### 2.8. Histomorphological and Omics Analyses

Within 45 min post-mortem, samples of the longissimus dorsi muscle were collected and trimmed into tissue blocks measuring 1.0 cm × 1.0 cm × 0.3 cm. These tissue blocks were immediately placed in a 10% neutral formalin solution for fixation for 48 h. After fixation, the tissue blocks were rinsed with running water for 4 h, followed by dehydration through a gradient of alcohol solutions (70%, 80%, 90%, 95%, and 100%), clearing with xylene, infiltration with paraffin, and embedding. The paraffin-embedded blocks were then sectioned into continuous 5 μm thick slices using a rotary microtome. These slices were mounted onto glass slides and baked overnight at 45 °C. After routine dewaxing and rehydration of the sections, hematoxylin–eosin (HE) staining was performed: the sections were stained with hematoxylin for 8 min, rinsed with running water for bluing, stained with eosin for 1 min, dehydrated through a gradient of alcohol solutions, cleared with xylene, and then mounted with neutral balsam [14]. The stained sections were subjected to whole-slide digital scanning using an automated slide scanner (Lise-Meitner-Str. 11-D-55129, Mainz, Germany) to obtain high-definition images for subsequent analysis.

### 2.9. Fatty Acid Determination

Mix the individual fatty acid single-standard reserve solutions, and prepare a series of calibration standards using a fat-free matrix. Precisely measure an appropriate amount of the isotope internal standard, and mix it to prepare the internal standard working solution. All the standards and the internal standard solutions are stored at −20 °C. Sample pretreatment: Take an appropriate amount of sample, add 150 μL methanol, 500 μL MTBE and 125 μL water, vortex mix and then let it stand at −20°C for 30 min. Centrifuge at 12,000 rpm for 10 min and take the supernatant. Add the extraction reagent to the lower layer and extract once again. Combine the two supernatants. Add 500 μL 0.4 M sodium hydroxide–methanol solution, and saponify at 70 °C for 30 min. After cooling, add 67 μL 3 M hydrochloric acid for acidification, mix and let it stand for 5 min, then centrifuge at 12,000 rpm for 10 min. Take the supernatant, blow dry with nitrogen, and re-dissolve with 100 μL 80% methanol. Take 50 μL of the re-solution, add 150 μL of derivatization reagent, derivatize at 40 °C for 40 min. Take 47.5 μL of the derivatized solution, add 2.5 μL of the mixed internal standard solution, mix, and then send for detection. The fatty acid quantification analysis was carried out using an ultra-high performance liquid chromatography–tandem mass spectrometry system (ExionLC™ AD UHPLC-QTRAP 6500, AB SCIEX, Framingham, MA, USA). The chromatographic column was a Waters ACQUITY UPLC BEH C18 column (2.1 mm × 100 mm, 1.7 μm), with a column temperature of 40 °C. The mobile phase A was 0.1% formic acid–acetonitrile/water (1:1), and the mobile phase B was isopropanol/acetonitrile (1:1). The flow rate was 0.30 mL/min. The gradient elution program was as follows: 0–1 min, 45% B; 1–5.5 min, 45–70% B; 5.5–14.5 min, 70–80% B; 14.5–15 min, 80–100% B; 15.1–17 min, 45% B. The mass spectrometry was conducted in the negative ion multiple reaction monitoring mode. Ion source parameters: Spray voltage—4500 V, curtain gas 35 psi, ion source temperature 550 °C; both the nebulizing gas and auxiliary gas were 60 psi [15].

### 2.10. Untargeted Muscle Metabolomics Analysis

#### 2.10.1. Metabolite Extraction

A 100 mg portion of muscle tissue ground in liquid nitrogen was taken, and 500 μL of 80% methanol–water solution was added. After vortexing and shaking, the mixture was allowed to stand on ice for 5 min. It was then centrifuged at 15,000× *g* for 20 min at 4 °C. The supernatant was collected and diluted with mass spectrometry-grade water to achieve a methanol content of 53%. Another round of centrifugation (4 °C, 15,000× *g*, 20 min) was carried out, and the resulting supernatant was collected for liquid chromatography–mass spectrometry (LC-MS) analysis.

#### 2.10.2. Liquid Chromatography–Mass Spectrometry Conditions

A Hypersil Gold C18 column (2.1 mm × 100 mm, 1.7 μm) was used, with a column temperature of 40 °C and a flow rate of 0.2 mL/min. Mobile phase A consisted of 0.1% formic acid in water, and mobile phase B was methanol. Mass spectrometry analysis was performed in both positive and negative ion modes, with a scanning range of *m*/*z* 100–1500. The ion source parameters were as follows: spray voltage of 3.5 kV, sheath gas flow rate of 35 psi, auxiliary gas flow rate of 10 L/min, ion transfer tube temperature of 320 °C, auxiliary gas heater temperature of 350 °C, and S-lens RF level of 60 [16]. Data-dependent scanning was employed to acquire secondary mass spectrometry data.

#### 2.10.3. Data Processing and Metabolite Identification

The raw data were converted into the mzXML format using ProteoWizard. Peak extraction, alignment, and quantification were carried out using XCMS. Metabolites were identified by comparison with Novogene’s in-house database (NovoMetDB), with a mass deviation set at 10 ppm. Metabolites with more than 50% missing values were filtered out. The remaining missing values were imputed using the k-nearest neighbors (KNN) algorithm, and background ions were removed. The raw quantification results were normalized using the formula (raw quantification value of the sample/(sum of quantification values of metabolites in the sample/sum of quantification values of metabolites in quality control (QC) samples)). Metabolites with a relative peak area coefficient of variation (CV) > 30% in QC samples were excluded [17]. The identified metabolites were annotated using the Kyoto Encyclopedia of Genes and Genomes (KEGG), Human Metabolome Database (HMDB), and LIPID Maps databases.

Principal component analysis (PCA) and partial least squares discriminant analysis (PLS-DA) were conducted using metaX [18] software to calculate the variable importance in the projection (VIP) values. Univariate analysis was performed using the *t*-test to compare differences between groups, and the fold change (FC) was calculated. The criteria for screening differential metabolites were VIP > 1, *p* < 0.05, and |log2FC| > 0 [19]. The volcano plot was drawn using the R package ggplot2, and the cluster heatmap was generated using Pheatmap. Metabolic pathway enrichment analysis was based on the KEGG database, and the hypergeometric test was used to determine significantly enriched pathways (*p* < 0.05).

### 2.11. 16S rRNA Sequencing Analysis

#### 2.11.1. DNA Extraction and PCR Amplification

Total DNA was extracted from the samples using the CTAB method. With the extracted DNA as the template, the V4 region of the bacterial 16S rRNA gene was amplified using the primers 515F (5′-GTGCCAGCMGCCGCGGTAA-3′) and 806R (5′-GGACTACHVGGGTWTCTAAT-3′). The PCR reaction system (15 μL) consisted of 10 ng of DNA template, 0.2 μM of each primer, and 1× Phusion High-Fidelity PCR Master Mix. The amplification program was as follows: initial denaturation at 98 °C for 1 min; 30 cycles of denaturation at 98 °C for 10 s, annealing at 50 °C for 30 s, and extension at 72 °C for 30 s; and a final extension at 72 °C for 5 min. After purification, the PCR products were used to construct a library.

#### 2.11.2. Sequencing and Data Processing

The library was sequenced on the Illumina NovaSeq 6000 platform using paired-end sequencing. The raw sequencing data were split according to the barcodes. The paired-end reads were merged using FLASH, and quality control and filtering were performed using fastp. Chimeric sequences were removed by comparing the sequences with the Silva database (v138.1) using the UCHIME algorithm, resulting in valid data. Operational taxonomic units (OTUs) were generated by clustering the sequences with a 97% similarity threshold using Uparse (v7.0. 1001) software, and representative sequences were selected and compared with the Silva 138.1 database for species annotation.

#### 2.11.3. Bioinformatics Analysis

Alpha diversity was calculated using Qiime (Version1.9.1) software to determine indices such as Chao1, Shannon, and Simpson. Beta diversity was analyzed based on UniFrac distances, and principal coordinate analysis (PCoA) and non-metric multidimensional scaling (NMDS) were performed. Differences between groups were tested using Anosim and Adonis tests, and LEfSe was used to screen for biomarkers (LDA > 2).

### 2.12. Statistical Analysis

The experimental data were initially organized using Excel 2016 software (Microsoft Corp., USA), and then analyzed using SPSS 20.0 software (IBM Corp., Armonk, NY, USA). Data on growth performance (Table 2) were analyzed using a one-way ANOVA with pen as the experimental unit. Slaughter performance, meat quality, fatty acid, metabolomic (relative abundance), and microbiota data from the CK vs. MS60 comparison were analyzed using an independent samples *t*-test. For all ANOVAs, post hoc comparisons were performed using the LSD method. Metabolomics and microbiota Beta-diversity significance was assessed using PERMANOVA (Adonis). The results were expressed as “mean ± standard deviation”, and *p* < 0.05 indicated a significant difference, while *p* < 0.01 indicated an extremely significant difference.

## 3. Results

### 3.1. Effects of Feeding Cotton Stalk–Beet Pulp Microsilage on Weight Gain Performance of Suffolk Rams

As shown in Table 2, on the 120th day of the experiment, the final body weights and net weight gains of all experimental groups (MS30, MS60, and MS90) were significantly higher than those of the CK group (*p* < 0.01). Among them, the final body weight and net weight gain of the MS60 group were significantly higher than those of the CK and MS90 groups (*p* < 0.05), while showing no significant difference from the MS30 group (*p* > 0.05). The average daily feed intake of the MS60 group was significantly higher than that of the CK group (*p* < 0.01) and also significantly higher than those of the MS30 and MS90 groups (*p* < 0.05). Both the MS30 and MS60 groups had significantly higher average daily feed intakes compared to the CK group (*p* < 0.01), with no significant difference observed between the MS30 and MS60 groups (*p* > 0.05). The feed-to-gain ratio of the CK group was significantly higher than those of the MS30 and MS60 groups (*p* < 0.01) and also significantly higher than that of the MS90 group (*p* < 0.05). The feed-to-gain ratios of the MS30 and MS60 groups were significantly lower than that of the MS90 group (*p* < 0.05), with no significant difference between the MS30 and MS60 groups (*p* > 0.05).

**Table 2:** Effects of feeding cotton stalk–beet pulp microsilage on weight gain performance of Suffolk rams.

Item	Group				*p*-Value
	CK	MS30	MS60	MS90	
Initial body weight, kg	32.33 ± 3.57	32.22 ± 1.01	32.47 ± 1.55	32.42 ± 5.55	0.999
Final body weight, kg	54.09 ± 1.71 ^C^	61.13 ± 2.97 ^A^	62.58 ± 2.81 ^A^	56.96 ± 3.12 ^B^	0.001
Net weight gain, kg	21.76 ± 2.04 ^C^	28.91 ± 2.73 ^A^	30.11 ± 2.06 ^A^	24.54 ± 3.84 ^B^	0.001
Average daily feed intake, kg/d	1.52 ± 0.01 ^C^	1.59 ± 0.02 ^B^	1.65 ± 0.02 ^A^	1.63 ± 0.02 ^B^	0.001
Feed conversion ratio	8.45 ± 0.82 ^A^	6.66 ± 0.66 ^C^	6.58 ± 0.41 ^C^	8.13 ± 1.17 ^B^	0.001

Note: Data are expressed as mean ± standard deviation. In the same row, different uppercase letters indicate highly significant differences (*p* < 0.01).

### 3.2. Effects of Feeding Cotton Stalk–Beet Pulp Microsilage on Meat Production Performance of Suffolk Rams

As shown in Table 3, the pre-slaughter live weight and carcass weight of the MS60 group were significantly higher than those of the CK group (*p* < 0.01). The dressing percentage and the weight of head and hooves in the MS60 group were significantly higher than those in the CK group (*p* < 0.05). However, there were no significant differences in the eye muscle area and backfat thickness between the MS60 and CK groups (*p* > 0.05).

### 3.3. Effects of Feeding Cotton Stalk–Beet Pulp Microsilage on Meat Quality of Suffolk Rams

As indicated in Table 4, the shear force of the MS60 group was significantly lower than that of the CK group (*p* < 0.01). The crude fat content, meat color lightness (L*), and redness (a*) of the MS60 group were significantly higher than those of the CK group (*p* < 0.05), while the meat color yellowness (b*) of the MS60 group was significantly lower than that of the CK group (*p* < 0.05). There were no significant differences in crude protein content, pH value, cooked meat percentage, or drip loss between the two groups (*p* > 0.05), although the MS60 group showed a tendency towards higher values.

**Figure 1:**
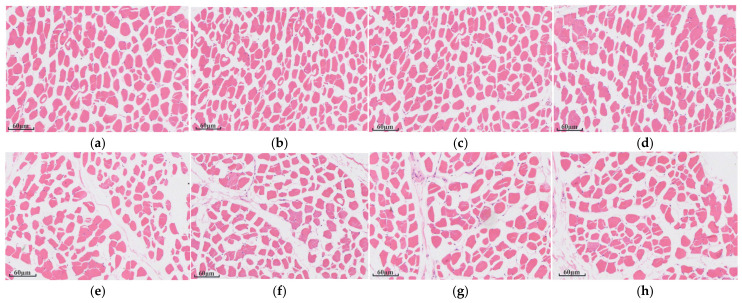
Histological sections of the longissimus dorsi muscle. (**a**–**d**) depict histological sections of the longissimus dorsi muscle from the CK group (control group fed a basal diet containing 21% whole-plant corn microsilage). (**e**–**h**) show histological sections of the longissimus dorsi muscle from the MS60 group (where 60% of the whole-plant corn microsilage in the basal diet was replaced with cotton stalk–beet pulp microsilage). In the images, pink tissue represents muscle fibers, white tissue indicates intramuscular fat (MIF), and blue represents cell nuclei. Magnification: 100×; scale bar = 60 μm. Visual assessment indicates a larger area of intramuscular fat deposition (white tissue) in the MS60 group compared to the CK group.

Figure 1 illustrates that the adipose cells in the HE-stained sections of the longissimus dorsi muscle have well-defined boundaries. The intramuscular fat deposition in the MS60 group was notably greater than that in the CK group.

### 3.4. Effects of Feeding a Cotton Stalk–Beet Pulp Mixed Microsilage Feed on the Fatty Acid Content in the Longissimus Dorsi Muscle of Suffolk Rams

As indicated in Table 5, compared with the CK group, the MS60 group exhibited a significantly higher content of total fatty acids (TFAs^6^) (*p* < 0.05). In the MS60 group, the contents of caprylic acid (C8:0) and capric acid (C10:0) were highly significantly lower (*p* < 0.01), and the content of total saturated fatty acids (SFAs^1^) was also highly significantly lower (*p* < 0.01), while the content of pentadecanoic acid (C15:0) was highly significantly higher (*p* < 0.01). The content of total monounsaturated fatty acids (MUFAs^3^) in the MS60 group was highly significantly higher (*p* < 0.01), among which the content of erucic acid (C22:1n-9t) was highly significantly higher (*p* < 0.01), and the contents of myristoleic acid (C14:1n-5c), palmitoleic acid (C16:1n-7c), oleic acid (C18:1n-9c), cis-11-octadecenoic acid (C18:1n-7c), α-linolenic acid (C18:3n-3), trans-myristoleic acid (C14:1n-5t), and trans-10-pentadecenoic acid (C15:1n-5t) were significantly higher (*p* < 0.05). There were no significant differences between the two groups in the contents of myristic acid (C14:0), palmitic acid (C16:0), stearic acid (C18:0), linoleic acid (C18:2n-6c), arachidonic acid (C20:4n-6), EPA, DHA, elaidic acid (C18:1n-9t), trans-11-octadecenoic acid (C18:1n-7t), total SFA^2^, total polyunsaturated fatty acids (PUFA^4^), and total n-6 PUFAs (PUFAs^5^) (*p* > 0.05).

### 3.5. Effects of Feeding a Cotton Stalk–Beet Pulp Mixed Microsilage Feed on Metabolomics in the Longissimus Dorsi Muscle of Suffolk Rams

#### 3.5.1. Principal Component Analysis

**Figure 2:**
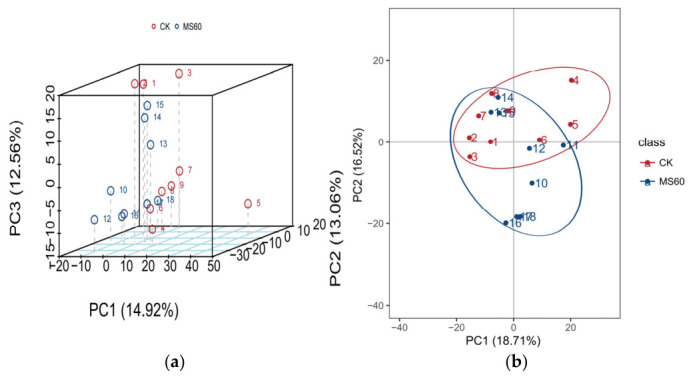
Principal component analysis (PCA) between groups. (**a**) presents the 3D plot of PCA between groups, while (**b**) shows the score plot of PCA between groups. In the figures, the *x*-axis (PC1) and *y*-axis (PC2) represent the scores of the first- and second-ranked principal components, respectively. Scatter points of different colors denote samples from different experimental groups, and the ellipses represent the 95% confidence intervals (when the number of biological replicates is less than four, the 95% confidence ellipses cannot be displayed).

As shown in Figure 2a, the sample points from the CK and MS60 groups exhibit a significant separation trend along the first principal component. The variance contribution rates are 14.92% for PC1, 13.06% for PC2, and 12.56% for PC3. In the two-dimensional plot Figure 2b, the samples from the two groups fall within distinct 95% confidence ellipses, further verifying significant metabolic differences between the groups. This indicates that feeding intervention with the cotton stalk–beet pulp mixed microsilage feed can significantly alter the metabolism in the muscle tissue of Suffolk rams. Moreover, the differences between the groups are greater than the biological variation within the groups, confirming the reliability of the experimental grouping and treatment effects.

#### 3.5.2. OPLS-DA Results for Metabolites

**Figure 3:**
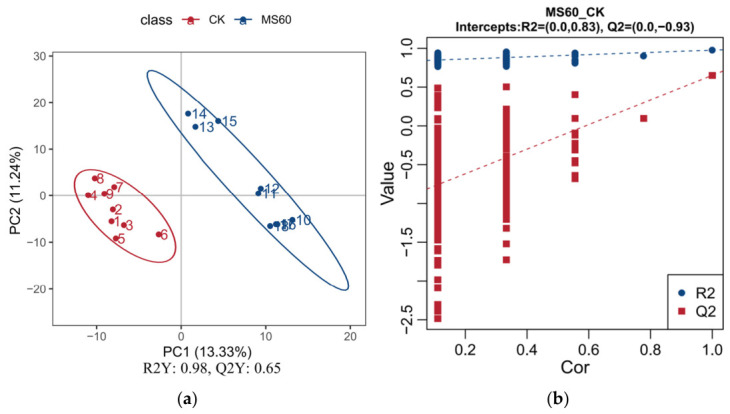
(**a**): OPLS-DA score plot, (**b**): OPLS-DA validation plot. The score scatter plot displays the scores of samples on the first principal component as the *x*-axis and on the second principal component as the *y*-axis. R2Y represents the explained variance ratio of the model, while Q2Y is used to evaluate the predictive ability of the PLS-DA (or here OPLS-DA, to be precise in context, though the original says PLS-DA, but we are discussing OPLS-DA, so we can adjust accordingly) model. A model is considered well-established when R2Y is greater than Q2Y.

To further enhance the discriminative power between groups and screen for key metabolites, supervised Orthogonal Projections to Latent Structures Discriminant Analysis (OPLS-DA) was conducted. As shown in Figure 3a, the OPLS-DA score plot clearly separates the samples from the CK group and the MS60 group, indicating that the model can effectively capture systematic metabolite variations associated with the feeding treatments. The model parameters demonstrate good explanatory power and predictive capability. The validation results from permutation tests (Figure 3b) confirm that the Q^2^ value of the original model is significantly higher than that of the models after random permutations. Moreover, the intercept of the regression line with the *Y*-axis is negative, proving that the established OPLS-DA model is robust and reliable without overfitting, and can be used for the reliable screening of differential metabolites in subsequent analyses.

#### 3.5.3. Screening Results of Differential Metabolites

**Figure 4:**
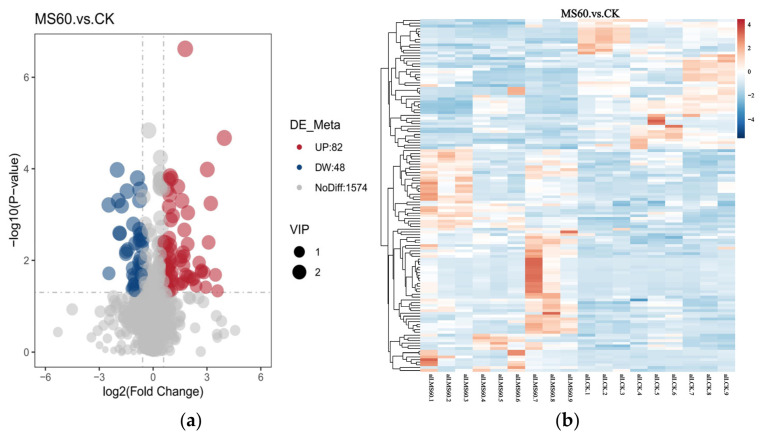
Analysis of differential metabolites between the MS60 and control (CK) groups. (**a**): Volcano plot. The x-axis represents the log_2_(fold change), and the y-axis represents the −log_10_(*p*-value). Each point represents a metabolite: red points indicate significantly upregulated metabolites (VIP > 1, *p* < 0.05, FC > 1.2), blue points indicate significantly downregulated metabolites (VIP > 1, *p* < 0.05, FC < 0.833), and gray points represent metabolites with no significant difference. The size of each point corresponds to its VIP value. (**b**): Rows represent differential metabolites, and columns represent samples. The color scale from blue to red indicates the relative expression level of metabolites from low to high. The clustering tree for metabolites is shown on the left, and the clustering tree for samples is shown on the top.

To comprehensively analyze the metabolic differences between the MS60 group and the CK group, multivariate statistical analysis combined with univariate analysis was employed to screen for differential metabolites. The screening thresholds were set as variable importance in the projection (VIP) > 1.0, fold change (FC) > 1.2 or FC < 0.833, and *p*-value < 0.05. From Figure 4a, a total of 206 differential metabolites were identified, of which 136 metabolites were significantly upregulated, and 70 metabolites were significantly downregulated. The heatmap analysis (Figure 4b) further validated these findings, revealing distinct clustering patterns among the differential metabolites.

#### 3.5.4. Metabolic Pathways of Differential Metabolites

**Figure 5:**
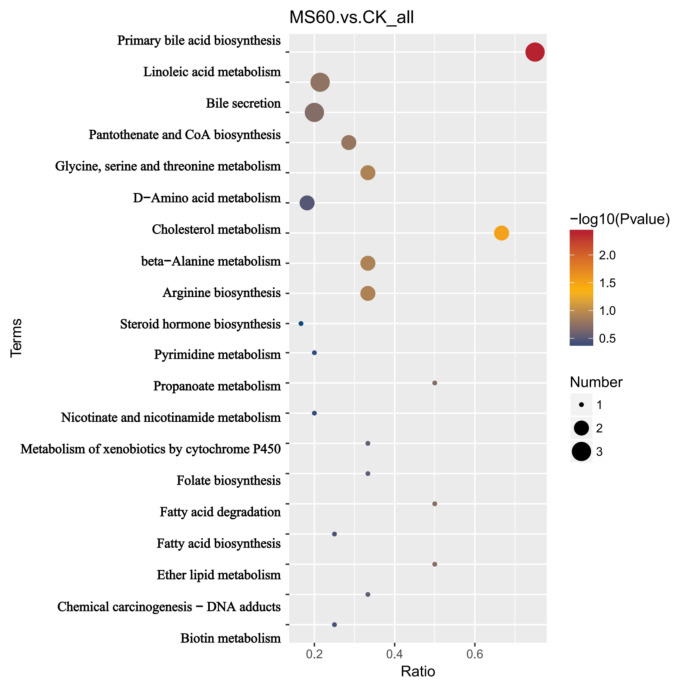
KEGG enrichment bubble plots. The *x*-axis represents the enrichment factor (number of differential metabolites/total number of metabolites in the pathway), indicating the degree of enrichment. The color represents the *p*-value from the hypergeometric test (the greener the color, the smaller the *p*-value and the more significant the enrichment). The size of the dots represents the number of differential metabolites in the pathway.

The KEGG enrichment analysis revealed that based on the differential metabolites between the MS60 group and the CK group, 20 metabolic pathways were significantly enriched (Figure 5). The most significantly enriched pathways included primary bile acid synthesis, linoleic acid metabolism, and bile secretion. Other enriched pathways mainly involved lipid metabolism, amino acid metabolism, and vitamin metabolism.

### 3.6. Effects of Feeding Cotton Stalk–Beet Pulp Mixed Microsilage on Jejunal Microbiota of Suffolk Rams

#### 3.6.1. Alpha Diversity Analysis

**Figure 6:**
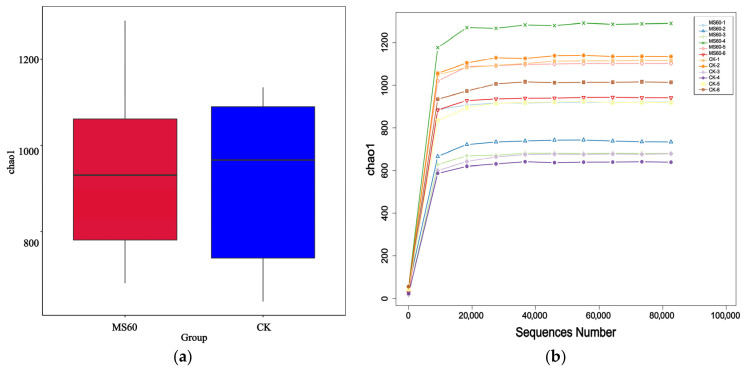
Alpha diversity analysis. (**a**) Box plot of Alpha diversity indices between groups; (**b**) species rarefaction curves (sequencing data volume—index curves). The flattening of the curves indicates that the sequencing depth is adequate.

Alpha diversity analysis was employed to evaluate the species richness and evenness of microbial communities within each group’s samples. The analysis was conducted using inter-group difference box plots (Figure 6a) and rarefaction curves (Figure 6b). The results indicated that there were no significant differences in the Shannon index and Chao1 index between the MS60 group and the CK group (*p* > 0.05). The rarefaction curves flattened out after reaching approximately 30,000 sequencing reads, suggesting that the current sequencing depth was sufficient to cover the vast majority of microbial species in the samples, thereby ensuring the reliability of subsequent analytical results.

#### 3.6.2. Beta Diversity Analysis

**Figure 7:**
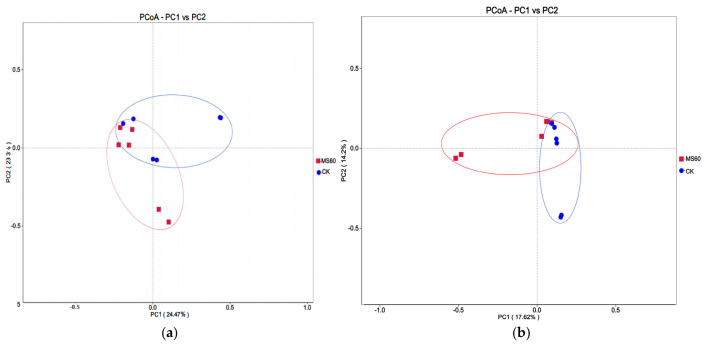
(**a**,**b**) are two-dimensional PCoA (Principal Coordinate Analysis) plots ((**a**): Weighted Unifrac distance; (**b**): Unweighted Unifrac distance). Beta diversity analysis was performed to assess differences in microbial community structure between groups.

Beta diversity analysis was conducted to evaluate differences in microbial community structure between groups. Principal Coordinate Analysis (PCoA) based on Weighted Unifrac and Unweighted Unifrac distances revealed (Figure 7a,b) that samples from the CK group and the MS60 group exhibited a clear separation trend in two-dimensional space, particularly in the Weighted UniFrac distance plot. This indicates significant differences in gut microbial community structure between the two groups, suggesting that feeding cotton stalk–beet pulp mixed microsilage significantly reshaped the gut microbiota composition of Suffolk rams.

#### 3.6.3. Analysis of Species Distribution at the Phylum and Genus Levels

**Figure 8:**
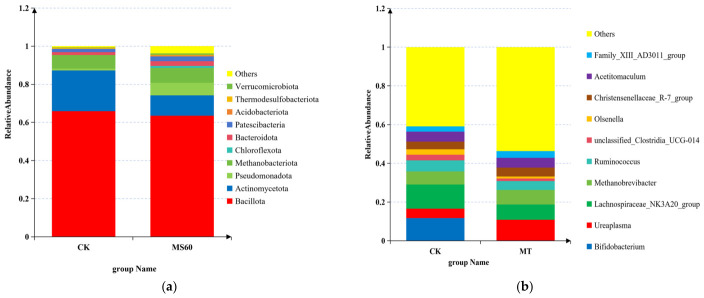
The 10 species with the highest relative abundance at the genus level and the phylum level. (**a**): Bar chart showing the top 10 species with relative abundance at the phylum level. (**b**): Bar chart showing the top 10 species with relative abundance at the genus level. The *x*-axis represents one principal coordinate, and the *y*-axis represents another principal coordinate. The percentages indicate the contribution of the principal coordinates to the sample differences. Each point in the figures represents a sample, and samples from the same group are depicted using the same color.

The composition and relative abundance of the ruminal microbiota were analyzed at both the phylum and genus levels (Figure 8).

At the phylum level (Figure 8a), no significant differences were observed in the overall microbial community structure between the CK and MS60 groups. The phyla Bacillota and Methanobacteriota maintained relatively high abundances in both groups, accounting for 65.91% and 7.13% in the CK group, and 63.51% and 7.78% in the MS60 group, respectively. Pseudomonadota exhibited an increasing trend in the MS60 group (0.95% and 6.64%), while the abundance of Patescibacteria was significantly higher in the MS60 group compared to the CK group (1.75% and 2.52%). These findings suggest that the dietary intervention may exert a selective regulatory effect on specific bacterial phyla.

At the genus level (Figure 8b), the relative abundance of *Bifidobacterium* was significantly lower in the MS60 group than in the CK group (*p* < 0.001). No significant differences were detected between the two groups for *Methanobrevibacter*, *Ruminococcus*, or the Christensenellaceae R-7 group. Notably, Bifidobacterium showed a higher abundance in the CK group (CK: 14.1%, MS60: 0.2%). Furthermore, the proportion of the “Others” category exhibited an increasing trend in the MS60 group (40.9% and 53.6%), indicating that the intervention may influence the compositional structure of low-abundance microbial taxa.

#### 3.6.4. LEfSe Analysis

**Figure 9:**
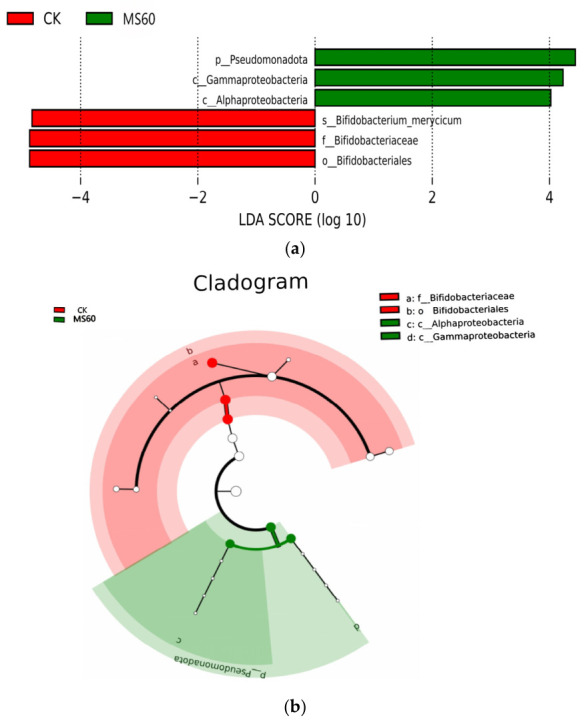
(**a**) Histogram of linear discriminant analysis (LDA) scores (LDA threshold > 4). The length of the bars represents the effect size of the differentially abundant taxa. (**b**) Cladogram. The concentric circles radiating from the inside out represent taxonomic levels from phylum to genus. The size of the nodes is proportional to the relative abundance. Color code: White, no significant difference; red, important taxa in the red group; green, important taxa in the green group; differential biomarkers follow group colors.

To further identify microbial taxa with significant differences between groups, LEfSe analysis was conducted (Figure 9a,b). The histogram of linear discriminant analysis (LDA) scores revealed that several genera, such as Romboutsia and Lactobacillus, in the MS60 group exhibited relatively high LDA scores (LDA > 4.0), indicating that these taxa were significantly enriched in the MS60 group. The cladogram visually illustrates the distribution of these differentially abundant taxa from a phylogenetic perspective. The analysis results suggest that feeding a mixed microsilage of cotton stalk–beet pulp may optimize the intestinal microecological structure of Suffolk rams by enriching specific probiotic populations (e.g., Lactobacillus) and suppressing certain potential opportunistic pathogens.

### 3.7. Correlation Analysis Between Jejunal Microbial Taxa at the Phylum Level and Muscle Fatty Acid Contents in Suffolk Rams

**Figure 10:**
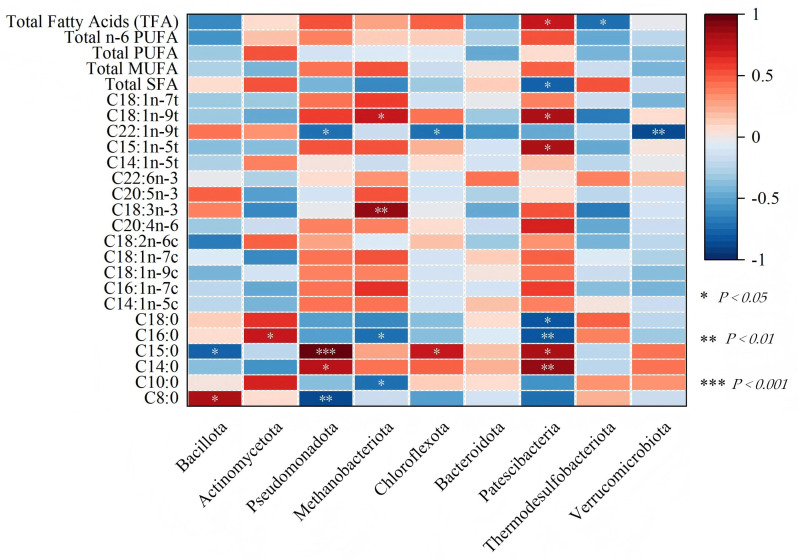
Correlation analysis heatmap of jejunal bacterial phyla and key fatty acid contents in the longissimus dorsi muscle of Suffolk rams. Correlation between major bacterial phyla in the jejunum of Suffolk rams and key fatty acid content in the longissimus dorsi muscle. The heatmap visually represents the magnitude and direction of Pearson correlation coefficients (r values) using a color gradient, where blue indicates negative correlation, and red indicates positive correlation. Darker colors denote stronger correlations. Significance levels are denoted by asterisks.

As shown in Figure 10, the phylum Bacillota exhibited a significant positive correlation with C8:0 (*p* < 0.05) and a significant negative correlation with C15:0 (*p* < 0.05). The phylum Actinomycetota showed a significant positive correlation with C16:0 (*p* < 0.05). The phylum Pseudomonadota had an extremely significant negative correlation with C8:0 (*p* < 0.001) and C15:0 (*p* < 0.01), and a significant positive correlation with C14:0 (*p* < 0.05). The phylum Methanobacteriota demonstrated an extremely significant positive correlation with C18:3n-3 fatty acid (*p* < 0.01), a significant positive correlation with C18:1n-9t (*p* < 0.05), and significant negative correlations with C10:0 and C16:0 (*p* < 0.05). The phylum Chloroflexota had a significant positive correlation with C15:0 (*p* < 0.05) and a significant negative correlation with C22:1n-9t (*p* < 0.05). The phylum Patescibacteria exhibited an extremely significant negative correlation with C14:0 (*p* < 0.01), significant positive correlations with C15:0, C15:1n-5t, C18:1n-9t, and total fatty acids (TFA) (all *p* < 0.05), an extremely significant negative correlation with C16:0 (*p* < 0.01), and a significant negative correlation with total saturated fatty acids (Total SFA) (*p* < 0.05). The phylum Thermodesulfobacteriota showed a significant negative correlation with TFA (*p* < 0.05). The phylum Verrucomicrobiota had an extremely significant negative correlation with C22:1n-9t (*p* < 0.01).

## 4. Discussion

### 4.1. Effects of Feeding a Mixed Microsilage of Cotton Stalk and Beet Pulp on the Weight Gain Performance of Suffolk Rams

In this study, the MS30 and MS60 groups demonstrated the best weight gain performance. Compared to the CK group, the MS30 group showed increases of 13.01% in final weight and 32.86% in net weight gain, while the MS60 group showed increases of 15.70% and 38.38%, respectively. These results are consistent with the conclusions of multiple studies that have utilized unconventional feeds to replace corn microsilage. For instance, Jianhua Yang et al. [20] found that feeding fermented cotton stalks to Hu sheep with replacement levels of 30% and 50% resulted in the highest final weights, with improved growth performance compared to the control group. Guo Tongjun et al. [21] reported that the feeding effect of steam-exploded and fermented cotton stalks was intermediate between that of corn microsilage and untreated cotton stalks. Wang Jiao et al. [22], in their study on feeding a mixed silage of sweet sorghum and licorice stems and leaves to Karakul sheep, also observed that replacement levels of 50% and 25% led to the best performance. Similarly, Cao Xianhong et al. [23] demonstrated that in beef cattle fed a mixed silage of forage mulberry and stevia residue, the groups with 20% and 40% replacement had significantly higher final weights and average daily gains than the control group. Collectively, these studies indicate that an appropriate proportion of unconventional feed replacement (30–60%) can partially substitute for corn microsilage without compromising production performance.

However, when the replacement ratio was increased to 90%, the weight gain performance of Suffolk rams significantly declined. Notably, the dry matter intake in the MS90 group (1.63 kg/d) was similar to that in the MS60 group (1.65 kg/d), indicating that reduced feed intake was not the primary cause of the decline in growth performance. This decline is likely attributed to a fundamental change in diet structure, where the higher replacement level may have increased the content of less digestible fiber, potentially reducing dry matter intake. Dadashi et al. [24] reported that sheep fed corn silage had significantly higher digestibility of dry matter and neutral detergent fiber than those fed silage containing sugar beet pulp and wheat straw. Another study found that increasing the proportion of cotton stalks in sheep diets significantly reduced the apparent digestibility of dry matter, organic matter, and neutral detergent fiber, while dry matter intake remained unchanged [25]. Therefore, the decline in growth performance in the 90% replacement group is more likely attributable to reduced energy digestibility rather than decreased dry matter intake. However, this inference still would require validation through apparent digestibility trials. In conclusion, under the experimental conditions of this study, the appropriate replacement ratio of the mixed microsilage of cotton stalk and beet pulp for corn microsilage is 30–60%, which can effectively integrate unconventional feed resources while ensuring the weight gain performance of meat sheep.

### 4.2. Effects of Feeding Cotton Stalk–Beet Pulp Mixed Microsilage on Meat Production Performance of Suffolk Rams

Slaughter performance is an important indicator for evaluating the meat production capacity of livestock and the economic benefits of breeding. The results of this study show that the MS60 group significantly increased the pre-slaughter live weight, carcass weight, slaughter rate, and head and hoof weight of Suffolk rams. This result is consistent with the previous trend of improved growth performance, indicating that the improvement in slaughter performance is closely related to the increase in pre-slaughter live weight [26]. In this study, the slaughter rate of the MS60 group (51.96%) was significantly higher than that of the CK group (50.10%), which is similar to the research results of Yuan Kang [27] and others on meat rabbits. This study also found that fermented beet pulp can increase the slaughter rate of meat rabbits. Wang Jiao et al. [22] found that the mixed silage of sweet sorghum and licorice stems and leaves significantly improved the slaughter performance, carcass weight, and dressing percentage of Karakul sheep, indicating that mixed microsilage of non-traditional forage resources can effectively enhance meat production performance in ruminants. Microbial fermentation of crop by-products can effectively improve their nutritional value and thereby enhance slaughter performance. Cheng Siyuan et al. [28] in the study on sweet beet sugar–malt fermented feed for meat sheep found that the fermented feed can improve the slaughter performance of meat sheep, and the results of this study are consistent with this. Zhu Yanhua et al. [29] found in their research that the mixed feed of cotton stalks and beet pulp, treated by compound micro-fermentation, has great advantages in improving the slaughter performance of Hu sheep. This may be because the metabolic products produced during fermentation or microsilage have positive effects on promoting the synthesis metabolism and improving the net meat ratio in different species.

In terms of eye muscle area and backfat thickness, there were no significant differences among the groups, but the MS60 group showed a slightly increased trend. The mixed microsilage feed of cotton stalks and beet pulp, under the substitution ratio of MS60, can significantly improve the key meat production performance indicators (carcass weight and slaughter rate) while promoting overall growth, and does not have a negative impact on eye muscle area and backfat thickness, achieving a synergistic improvement in growth performance and meat production performance, and is a type of coarse feed utilization with application value.

### 4.3. Effects of Feeding Cotton Stalk–Beet Pulp Mixed Microsilage on Lamb Meat Quality in Suffolk Rams

The quality of mutton is mainly determined by the contents of protein, fat, and water. Among them, the fat content has a significant impact on the juiciness, flavor, and tenderness of the meat, while the protein content directly affects the nutritional value of the meat [30]. In this experiment, the shear force of the MS60 group was significantly lower than that of the CK group, indicating that the muscles in the MS60 group were tender and juicy. Shear force is a key indicator for measuring the tenderness of muscles, and the lower the value, the more tender and juicy the meat is. Zhao Ran [31] found in a study on the effects of different feed types on pork quality that feeding liquid fermented feed could significantly reduce the shear force of pork and improve the tenderness of pork. The results of this study are consistent with this. Studies have shown that the intramuscular fat content has a significant impact on the differences in shear force between specific muscle groups [32]. In this study, the crude fat content, intramuscular fat deposition, and lightness (L) and redness (a) of the MS60 group were significantly higher than those of the CK group. Zhu Kai et al. [33] reported that replacing 70% of the basal diet with 30% fermented tail vegetable feed significantly reduced the shear force and pH value of the longissimus dorsi muscle, while significantly increasing lightness, redness, and marbling score, thereby improving meat tenderness and overall quality. Wang Hua et al. [34] found that microsilage could increase the intramuscular fat deposition of Hu sheep.

Color is an important indicator for evaluating the freshness of meat quality. Generally, the higher the redness value, the lower the brightness and yellowness values, and the more lustrous the meat color is [35]. After the slaughter of sheep, the initial pH value of the muscle is usually in the range of 6.0 to 7.0, and it will drop to 5.4 to 5.6 within 1 h after slaughter [36]. The rate of muscle pH value decline is closely related to the progress of various biochemical reactions. The faster the rate of muscle pH value decline, the faster the biochemical reactions such as protein denaturation [37], enzymatic reactions and microbial proliferation in the muscle will proceed [38]. Conversely, if the rate of muscle pH value decline is slower, the rate of these biochemical reactions will also decrease accordingly. The results of this experiment showed that there was no significant difference in pH values between the MS60 group and the CK group, and the pH values of both groups were within the normal range. The mixed microsilage of cotton stalks and beet pulp as a substitute for corn microsilage feeding to Suffolk rams can significantly improve the nutritional quality of mutton by increasing intramuscular fat content and protein deposition potential, optimizing meat color parameters, and reducing shear force. This indicates that microbial fermentation of agricultural by-products can effectively improve feed palatability and feed intake, thereby increasing dressing percentage and improving meat quality in ruminants.

### 4.4. The Effect of Feeding Cotton Stalk–Beet Pulp Mixed Microsilage on the Fatty Acid Content in the Longest Back Muscle of Suffolk Rams

Short-chain fatty acids (SCFAs) are mainly produced by rumen microorganisms through the fermentation of carbohydrates. They usually account for a relatively low proportion in the adipose tissue of ruminants, but excessive levels are often associated with a specific pungent odor [39]. This study found that the content of caprylic acid (C8:0) and capric acid (C10:0) in the muscles of the MS60 group was significantly lower than that of the CK group. This indicates that after the partial degradation of the lignocellulose in cotton stalk through fermentation, it may have changed the rumen microbial community structure [40], thereby reducing the deposition of these short-chain saturated fatty acids in the muscles [41]. At the same time, the total saturated fatty acid (SFA) content in the MS60 group was significantly lower than that in the CK group. Although there was no significant difference in the main long-chain SFAs such as tetradecanoic acid (C14:0), exadecenoic acid (C16:0), and octadecanoic acid (C18:0) between the two groups, the content of C18:0 showed a downward trend, which further optimized the nutritional structure of fatty acids.

Monounsaturated fatty acids (MUFAs) in ruminants mainly originate from two pathways: one is the direct deposition of unsaturated fatty acids in the diet; the other is the biological hydrogenation (Biohydrogenation) of dietary fats by rumen microorganisms and the endogenous conversion of saturated fatty acids by stearoyl-CoA desaturase (SCD) in muscle tissues, according to Cheng Pan [42]. In this experiment, the total MUFA content in the MS60 group was significantly higher than that in the CK group. The contents of pentadecanoic acid (C15:0), myristoleic acid (C14:1n-5c), and brazileic acid (C22:1n-9t) increased significantly, while the contents of palmitoleic acid (C16:1n-7c), oleic acid (C18:1n-9c), and cis-11-octadecenoic acid (C18:1n-7c) increased significantly. This result is similar to the finding of Bing Wang et al. [43] that adding tomato residue to the diet can increase the content of unsaturated fatty acids such as oleic acid in beef, and is also similar to the report of Zhan Cong et al. [44] that fermentation of goji residue has a significant effect on the content of oleic acid in the muscle of Tan sheep. Studies have shown that the content of palmitoleic acid (C16:1) in Mongolian sheep meat is significantly correlated with the flavor score [45]; the improvement of the flavor of Sunnite sheep meat is directly related to the Increase in the content of oleic acid (C18:1), while the accumulation of stearic acid (C18:0) will aggravate the pungent flavor [46]. The significant increase in oleic acid (C18:1n-9c) and palmitoleic acid (C16:1n-7c) in the MS60 group may be due to the large amount of propionic acid produced by the fermentation of soluble sugars and pectin in the cotton stalk–beet pulp mixed microsilage. Propionic acid, as a precursor for gluconeogenesis, can promote the generation of acetyl-CoA, providing a carbon skeleton for fatty acid synthesis. This study observed that the total saturated fatty acid (SFA) content in the muscles of the MS60 group was significantly reduced, while the total monounsaturated fatty acid (MUFA) content was significantly increased. Excessive intake of SFAs (especially tetradecanoic acid and hexadecanoic acid) is positively correlated with the risk of cardiovascular diseases, while MUFAs (such as oleic acid) have been proven to have the effect of lowering blood lipids and protecting the cardiovascular system [47]. Palmitoleic acid (C16:1n-7c) and oleic acid (C18:1n-9c) are important contributing substances to the characteristic flavor of sheep meat, and their content increase is positively correlated with the flavor score. The significant increase of these two MUFAs in this experiment suggests that the cotton stalk–beet pulp mixed microsilage feed may improve the eating quality of sheep meat by regulating fatty acid metabolism. Furthermore, the reduction of SFAs and the increase of MUFAs jointly optimized the fatty acid composition of the muscles, making it more in line with the demands of modern consumers for “healthy meat”. It is worth noting that the total fatty acid (TFA) content in the MS60 group was significantly higher than that in the CK group, which might lead to an increase in intramuscular fat (IMF) content, indicating that the mixed microsilage feed has certain effects in promoting lipid deposition.

### 4.5. Effects of Feeding a Mixed Microsilage of Cotton Stalk and Beet Pulp on the Metabolism of Longissimus Dorsi Muscle in Suffolk Rams

Given that the fatty acid content in the dorsal muscle is closely related to its metabolism, we further employed metabolomics analysis based on the observed changes in fatty acid composition to reveal the underlying metabolic pathways. Multivariate statistical analysis (PCA and OPLS-DA) indicated that the metabolic levels of the MS60 group were significantly separated from those of the CK group. The model’s explanatory rate and predictive ability were good (R2Y > Q2Y), suggesting that the feed intervention led to the overall metabolic remodeling of muscle tissue. KEGG pathway enrichment analysis identified 206 significantly different metabolites, mainly concentrated in lipid, amino acid, and energy metabolism-related pathways. The “2-oxoacid metabolism” pathway was significantly upregulated in the MS60 group. 2-oxo carboxylic acids are key intermediate products of the tricarboxylic acid cycle, indicating that the oxidative metabolic capacity of muscle mitochondria has been enhanced. The mixed microsilage of cotton stalk and beet pulp with high fiber and carbohydrates undergoes rapid fermentation in the rumen, promoting an increase in propionic acid production. Propionate is a key precursor for gluconeogenesis in ruminants and is converted to glucose in the liver to provide energy for peripheral tissues. Changes in energy metabolism may drive the enhancement of 2-oxo acid metabolism in muscles, providing energy and material basis for intramuscular fat deposition and fatty acid synthesis [48]. The “primary bile acid biosynthesis” pathway was significantly upregulated, while the “linoleic acid metabolism”, “arachidonic acid metabolism” and “biosynthesis of unsaturated fatty acids” pathways were significantly enriched. Bile acids can emulsify lipids, accelerate lipid digestion and absorption, and improve the efficiency of nutrient utilization [49]. The bile acid metabolism in this study was significantly activated, which may be related to the remodeling of the intestinal microbiota structure by mixed microsilage, promoting bile acid synthesis and reabsorption. The enhancement of bile acid signaling may inhibit excessive lipogenesis in the liver [50], and in muscle tissue, it may enhance the activity of stearoyl-CoA desaturase, promoting fatty acid desaturation, resulting in the accumulation of monounsaturated fatty acids, which is consistent with the previous results [51]. In addition, the enrichment of the “glycerophospholipid metabolism” pathway suggests the remodeling of cell membrane phospholipids. An increase in the proportion of oleic acid in membrane phospholipids can enhance the fluidity of the cell membrane, facilitating post-mortem muscle protein hydrolysis and improving meat tenderness [52]. The MS60 group significantly upregulated the “arginine biosynthesis” and “valine, leucine, and isoleucine (branched-chain amino acids, BCAAs) biosynthesis” pathways in the muscle of mutton. Arginine, as a precursor of nitric oxide, can dilate blood vessels and improve muscle nutrient supply [53]; the enhancement of BCAA synthesis promotes protein deposition, and its degradation products are the key precursors of the characteristic flavor of mutton [54]. The enrichment of the “protein digestion and absorption” pathway indicates that the MS60 feed has improved nitrogen utilization efficiency. In conclusion, the cotton stalk–beet pulp mixed microsilage feed significantly alters the metabolic pathway of the longissimus dorsi muscle in Suffolk rams through a multi-dimensional metabolic regulatory mechanism, thereby providing a mechanistic basis for the improved meat tenderness (reduced shear force) and the optimized fatty acid profile (increased MUFAs and decreased SFAs) observed in the MS60 group as described above.

### 4.6. Effects of Feeding a Mixed Microsilage of Cotton Stalk and Beet Pulp on the Jejunal Microbiota of Suffolk Rams

The results of this study indicate that a 60% proportion mixture of microsilage did not change the Alpha diversity of the small intestine microbiota, but significantly reshaped the Beta diversity, and exerted selective regulation on specific functional bacterial communities. This finding is consistent with the observation by Perea et al. [55] that feed efficiency phenotypes in lambs are associated with changes in the jejunal and colonic microbiota, highlighting the role of the small intestine in host metabolic regulation. In this study, there were no significant differences in the Shannon index and Chao1 index between the MS60 group and the CK group, indicating that the differences in microbial diversity between the two groups were relatively small. The microsilage mixture can replace part of the whole-plant corn microsilage in the diet, which is consistent with the research results of Ao Weiping [56]. The Beta diversity analysis results show that the 60% cotton stalk–beet pulp microsilage substitution treatment can significantly reshape the intestinal microbiota community structure of Suffolk rams, and the differences in the relative abundance of the bacterial community are the key factors driving the community differentiation between the groups. This is similar to the research conclusion of Cheng Ximing [57]. In this experiment, at the phylum level, there was no significant difference in the overall community structure between the CK group and the MS60 group. The Bacillota phylum is the core functional group for the degradation of carbohydrates and lipids in ruminant intestines [58], and the abundance of this phylum in this study is consistent with the research results of Elie, Jami [59] in dairy cows. The research of Henderson et al. [60] worldwide also confirmed that although there are significant differences in diet and hosts, the Bacillota phylum is always the core dominant bacterial group in the rumen gastrointestinal tract. At the genus level, the relative abundance of the Bifidobacterium genus in the MS60 group was significantly lower than that in the CK group. Dias et al. [61] found that the colonization of Bifidobacterium in the digestive tract of young ruminants is significantly affected by age and diet factors. The significant decrease in Bifidobacterium abundance in this experiment suggests that the cotton stalk–beet pulp microsilage may have changed the intestinal microecological balance and affected the colonization of beneficial bacteria. Methanobrevibacter genus, Ruminococcus genus, and the Christensenellaceae R-7 group in the two groups did not show significant differences. This is consistent with the research results of Rebecca D [62] on the stability of methanogenic archaea. This study further indicates that the microsilage mixture of cotton stalk–beet pulp does not cause significant fluctuations in the core gut microbiota and maintains the stability of the microbial community.

### 4.7. Correlation Analysis Between Jejunal Microbiota at the Phylum Level and Muscle Fatty Acid Content in Suffolk Rams

The correlation analysis revealed several associations between the jejunal microbiota and the fatty acid composition of muscle, providing mechanistic insights into the improved meat quality observed in the MS60 group. This study focuses on those associations that are most likely biologically meaningful and consistent with the phenotypic differences between the MS60 and CK groups. The abundance of Patescibacteria was significantly higher in the MS60 group than in the CK group. Correlation analysis showed that Patescibacteria was significantly positively correlated with monounsaturated fatty acids (e.g., C18:1n-9t) and total fatty acids, and significantly negatively correlated with total saturated fatty acids. The Patescibacteria may promote fatty acid desaturation by upregulating stearoyl-CoA desaturase (SCD) activity in muscle tissue, thereby increasing oleic acid content and enhancing membrane fluidity, which facilitates post-mortem proteolysis and improves meat tenderness. This requires experimental validation using gnotobiotic animal models or in vitro fermentation studies. Bacillota was positively correlated with caproic acid (C8:0) and negatively correlated with pentadecanoic acid (C15:0). Increased abundance of Bacillota promotes fat absorption and deposition [63], and its enhanced carbohydrate fermentation activity may increase medium-chain saturated fatty acid content while potentially inhibiting odd-chain fatty acid synthesis through modulation of propionate metabolism.

Actinomycetota was positively correlated with palmitic acid (C16:0), consistent with the report by Li et al. [64] that increased Actinomycetota abundance is associated with improved lipid metabolism. We speculate that Actinomycetota may enhance de novo lipogenesis in muscle tissue by providing acetyl-CoA precursors through enhanced fiber degradation, thereby supporting intramuscular fat deposition observed in the MS60 group. Methanobacteriota was positively correlated with α-linolenic acid (C18:3n-3) and trans-octadecenoic acid (C18:1n-9t), and negatively correlated with capric acid (C10:0) and palmitic acid (C16:0). Since methanogens are known to play a key role in hydrogen disposal and redox balance in the ruminant gut, these associations are likely indirect and may be mediated through alterations in gut redox potential affecting fatty acid biohydrogenation pathways. This requires further investigation. The enrichment of C18:3n-3 may contribute to the improved fatty acid profile and potential health benefits of MS60 lamb meat, though this requires further investigation. Verrucomicrobiota was negatively correlated with the long-chain trans fatty acid C22:1n-9t; it has been shown to support intestinal barrier function and immune regulation [64], and increased abundance may reduce the deposition of harmful long-chain trans fatty acids, thereby improving the nutritional quality of mutton in the MS60 group. Thermodesulfobacteriota was negatively correlated with total fatty acids, possibly influencing trans fatty acid conversion by affecting redox potential in the intestinal environment. In summary, these correlation-based findings are hypothesis-generating rather than confirmatory. They lay the foundation for future targeted experiments (e.g., fecal microbiota transplantation, selective inhibition of specific bacterial groups, or in vitro fermentation systems) to determine the causal relationships between specific microbial taxa and fatty acid metabolism in sheep. Moreover, these microbiota-driven changes in fatty acid metabolism provide a mechanistic basis for the observed reduction in shear force, increased intramuscular fat content, and optimized meat color in the MS60 group, linking jejunal microbial remodeling to the phenotypic improvements in meat quality and nutritional value.

## 5. Conclusions

The results of this study indicate that, compared with 0%, 30%, and 90% replacement levels, replacing 60% of whole-plant corn microsilage with cotton stalk–beet pulp mixed microsilage significantly improved the final body weight, net weight gain, and feed conversion ratio of Suffolk rams. In terms of meat quality and mechanisms, compared with the control group (CK), the 60% replacement level improved meat tenderness (reduced shear force), increased intramuscular fat content, optimized the fatty acid profile (decreased saturated fatty acids and increased monounsaturated fatty acids), and altered the muscle metabolome and jejunal microbiota. In practical production, the 60% replacement level is recommended to fully utilize the abundant local resources of cotton stalks and sugar beet pulp, alleviate feed shortages, and reduce production costs. This study provides a theoretical basis for developing new environmentally friendly feed, promoting the efficient utilization of agricultural by-products, and promoting the green and sustainable development of the sheep meat industry. In the future, further research can be conducted to explore the impact of this feed on the deposition of flavor substances in lamb meat and verify its applicability in different breeds of sheep, in order to promote its industrial application.

### 5.1. Limitations

The limitations of this study: Due to resource optimization considerations, the slaughter and omics analyses were only conducted for the CK group and the MS60 group. The MS30 group and the MS90 group were not included in the in-depth analysis, which limited the comprehensive assessment of the dose–effect differences at different levels. Secondly, the study subjects only included a single breed and gender (Suffolk rams), which limited the generalizability of the research results to other breeds, genders, or physiological stages. Moreover, the metabolomics and microbiomics analyses were based on correlational data and could not establish a causal relationship between the changes in the microbial community and the host metabolic phenotype.

### 5.2. Future Outlook

Subsequent studies should include all alternative levels (MS30, MS60, MS90) for a systematic comparison, and verify the findings of this study in different production systems (such as different breeding environments and management conditions). At the same time, it is recommended to adopt targeted experimental methods such as sterile animal models, fecal microbiota transplantation, or specific strain intervention to clarify the causal regulatory mechanism between intestinal microbiota and muscle metabolism.

## Figures and Tables

**Table 1:** Diet formulation and nutritional levels (on a dry matter basis %).

Item	CK ^1^	MS30 ^1^	MS60 ^1^	MS90 ^1^
Ingredients% ^2^				
Whole-plant corn microsilage	21	15	8	2
Cotton stalk–beet pulp mixed microsilage	0	5	11	16
Alfalfa hay	6	6	6	6
Corn stover	13	13	13	13
Corn grain	31	32	33	33.5
Cottonseed meal	6	6	6	6
Soybean meal	9	9	9	9.5
Wheat bran	6	6	6	6
Premix ^3^	3	3	3	3
Ruminant concentrate ^4^	3	3	3	3
Sodium bicarbonate	1	1	1	1
Salt	1	1	1	1
Total	100	100	100	100
Nutrient levels ^5^				
Crude protein, %	13.68	13.72	13.93	13.81
Ether extract, %	4.76	4.54	4.34	4.17
Metabolizable energy, MJ/kg	8.1	8.23	8.37	8.5
Crude ash, %	8.18	8.75	8.86	8.12
Neutral detergent fiber, %	36.33	37.74	36.45	36.42
Acid detergent fiber, %	23.5	24.5	25.5	26.5
Calcium, %	0.73	0.72	0.76	0.79
Total phosphorus, %	0.38	0.38	0.38	0.38

^1^ CK: The control group was fed with the basic feed (which contained 21% whole corn microsilage); MS30 group: 30% of the whole corn microsilage in the basic feed was replaced with cotton stalk–beet pulp microsilage; MS60 group: 60% of the whole corn microsilage in the basic feed was replaced with cotton stalk–beet pulp microsilage; MS90 group: 90% of the whole corn microsilage in the basic feed was replaced with cotton stalk–beet pulp microsilage. There were no significant differences in crude protein content and metabolizable energy levels among the groups (*p* > 0.05). ^2^ All roughage used in the trial was uniformly provided by the experimental site, while all concentrates were procured from Xinjiang Tiankang Feed Co., Ltd. (Urumqi, China). ^3^ Premix formulation: Vitamin A 130,000–250,000 IU, Vitamin D3 40,000–100,000 IU, Vitamin E ≥ 1400 IU, Copper (basic copper chloride) 550–800 mg, Iron (ferrous sulfate) 1500–7000 mg, Manganese (manganese sulfate) 1100–3000 mg, Zinc (zinc sulfate) 800–2000 mg, Iodine (calcium iodate) 20–30 mg, Selenium 8–12 mg, Zinc 20–30 mg, Calcium 10–20%, Total Phosphorus 1.5%, Sodium Chloride 12–20%, Moisture ≤ 10.0%. ^4^ Ruminant Feed: A feed produced by fermenting extruded corn, cottonseed meal, and bran using a mixed culture of lactic acid bacteria (*Lactobacillus acidophilus*), brewer’s yeast (*Saccharomyces cerevisiae*), and *Bacillus subtilis*. ^5^ All nutritional levels are actual measured values.

**Table 3:** Effects of feeding cotton stalk–beet pulp microsilage on meat production performance of Suffolk rams.

Item	Group		*p*-Value
	CK	MS60	
Pre-slaughter live weight, kg	54.09 ± 1.33 ^B^	62.58 ± 2.81 ^A^	0.001
Carcass weight, kg	27.10 ± 0.72 ^B^	32.48 ± 1.83 ^A^	0.002
Dressing percentage, %	50.10 ± 0.21 ^b^	51.96 ± 1.12 ^a^	0.018
Head and hoof weight, kg	3.60 ± 0.11 ^b^	4.03 ± 0.22 ^a^	0.013
Eye muscle area, cm^2^	15.99 ± 0.08	16.45 ± 0.61	0.188
Backfat thickness, mm	0.97 ± 0.21	1.06 ± 0.06	0.419

Note: Data are expressed as mean ± standard deviation. In the same row, different lowercase letters indicate significant differences (*p* < 0.05), different uppercase letters indicate highly significant differences (*p* < 0.01). An independent samples *t*-test was performed for comparison between CK and MS60 (*n* = 9 per group).

**Table 4:** Effects of feeding cotton stalk–beet pulp microsilage on meat quality of Suffolk rams.

Item	Group		*p*-Value
	CK	MS60	
Crude protein content, %	19.345 ± 1.990	20.546 ± 0.328	0.091
Crude fat content, %	3.826 ± 0.968 ^b^	5.363 ± 0.425 ^a^	0.010
pH_45min_	6.593 ± 0.112	6.644 ± 0.151	0.305
pH_24h_	5.835 ± 0.188	5.899 ± 0.083	0.247
L*_24h_ ^1^	32.542 ± 3.426 ^b^	35.038 ± 3.143 ^a^	0.025
a*_24h_ ^2^	20.867 ± 2.833 ^b^	23.111 ± 3.527 ^a^	0.037
b*_24h_ ^3^	3.452 ± 1.616 ^a^	2.233 ± 1.424 ^b^	0.018
Shear force, N	53.026 ± 6.566 ^A^	43.534 ± 0.767 ^B^	0.002
Cooking loss, %	69.113 ± 4.099	69.185 ± 3.150	0.982
Drip loss, %	28.350 ± 3.335	28.495 ± 4.272	0.965

Note: Data are expressed as mean ± standard deviation. In the same row, different lowercase letters indicate significant differences (*p* < 0.05), different uppercase letters indicate highly significant differences (*p* < 0.01). ^1^ L*_24h_ represents the brightness value of the mutton measured 24 h after slaughter; ^2^ a*_24h_ represents the redness value measured 24 h after slaughter; ^3^ b*_24h_ represents the yellowness value measured 24 h after slaughter.

**Table 5:** Fatty acid composition (μg/g) in the longissimus dorsi muscle of Suffolk rams fed with different diets (CK vs. MS60).

Item	Group		*p*-Value
	CK	MS60	
Methyl caprylate (C8:0)	389.46 ± 37.28 ^A^	263.67 ± 62.88 ^B^	0.002
Methyl caprate (C10:0)	809.62 ± 105.22 ^A^	507.24 ± 134.80 ^B^	0.001
Methyl myristate (C14:0)	2261.95 ± 190.76	3058.35 ± 543.28	0.055
Methyl pentadecanoate (C15:0)	85.11 ± 2.25 ^B^	181.43 ± 8.04 ^A^	0.001
Methyl palmitate (C16:0)	5200.53 ± 1421.51	4055.78 ± 771.73	0.221
Methyl stearate (C18:0)	5907.95 ± 1413.75	3689.85 ± 1487.39	0.106
Methyl myristoleate (C14:1n-5c)	245.52 ± 30.85 ^B^	331.40 ± 43.83 ^A^	0.004
Methyl palmitoleate (C16:1n-7c)	1242.28 ± 118.03 ^b^	1704.96 ± 300.10 ^a^	0.023
Methyl oleate (C18:1n-9c)	2725.27 ± 520.55 ^b^	4524.76 ± 1101.79 ^a^	0.018
Methyl cis-11-octadecenoate (C18:1n-7c)	2568.79 ± 325.60 ^b^	4381.11 ± 1086.90 ^a^	0.018
Methyl linoleate (C18:2n-6c)	2590.01 ± 825.97	3081.55 ± 296.83	0.266
Methyl arachidonate (C20:4n-6)	2717.30 ± 577.38	3207.90 ± 435.14	0.227
Methylα-linolenate (C18:3n-3)	309.05 ± 41.98 ^b^	379.10 ± 51.57 ^a^	0.038
Methyl eicosapentaenoate (EPA,C20:5n-3)	367.64 ± 39.37	476.50 ± 85.34	0.051
Methyl docosahexaenoate (DHA,C22:6n-3)	77.42 ± 12.75	95.53 ± 19.00	0.094
Methyl myristelaidate (C14:1n-5t)	13.00 ± 2.20 ^b^	19.93 ± 6.13 ^a^	0.039
Methyl trans-10-pentadecenoate (C15:1n-5t)	14.75 ± 1.67 ^b^	18.57 ± 3.26 ^a^	0.035
Methyl brassidate (C22:1n-9t)	5.15 ± 0.68 ^B^	6.41 ± 0.65 ^A^	0.003
Methyl elaidate (C18:1n-9t)	225.05 ± 50.88	322.75 ± 63.78	0.061
Methyl trans-11-octadecenoate (C18:1n-7t)	474.77 ± 714.14	306.70 ± 104.41	0.630
Total SFAs ^1^	16,634.77 ± 1929.66 ^A^	14,164.22 ± 1693.53 ^B^	0.006
Total SFAs ^2^	15,967 ± 2985	14,976 ± 2334	0.421
Total MUFAs ^3^	6940 ± 1120 ^B^	11,471 ± 2820 ^A^	0.009
Total PUFAs ^4^	6743 ± 1855	8168 ± 1080	0.125
Total n-6 PUFAs ^5^	5914 ± 1550	6949 ± 870	0.182
Total Fatty Acids (TFAs) ^6^	29,650 ± 3220 ^b^	34,615 ± 4150 ^a^	0.045

Note: Data are expressed as mean ± standard deviation. In the same row, different lowercase letters indicate significant differences (*p* < 0.05), different uppercase letters indicate highly significant differences (*p* < 0.01). ^1^ Total SFAs: The sum of saturated fatty acids listed in the table and those not listed. Its mean and standard deviation are calculated from the raw data. ^2^ Total SFAs: Includes the sum of undecanoic acid, dodecanoic acid, tridecanoic acid, heptadecanoic acid, stearic acid, arachidonic acid, eicosapentaenoic acid, and tetracosanoic acid not listed in the table. Its mean and standard deviation are calculated from raw data. ^3^ Total MUFAs: The sum of all monounsaturated fatty acids (MUFAs) listed in the table. ^4^ Total PUFAs: The sum of all n-6 and n-3 polyunsaturated fatty acids (PUFAs). ^5^ Total n-6 PUFAs: The sum of linoleic acid, gamma-linolenic acid, cis-11,14-docosadienoic acid, gamma-linolenic acid, arachidonic acid, and cis-7,10,13,16-docosatetraenoic acid. ^6^ Total fatty acids: The sum of all individual fatty acids measured.

## Data Availability

None of the data were deposited in an official repository. The data are available from the corresponding author upon reasonable request.

## Abbreviations

The following abbreviations are used in this manuscript:

ADF	Acid Detergent Fiber
ADG	Average Daily Gain
BCAA	Branched-Chain Amino Acid
BW	Body Weight
CK	Control Group (fed with basal diet containing 21% whole-plant corn microsilage)
CP	Crude Protein
CS	Cotton Stalk
DHA	Docosahexaenoic Acid
DM	Dry Matter
DMI	Dry Matter Intake
EE	Ether Extract
EPA	Eicosapentaenoic Acid
FC	Fold Change
HE	Hematoxylin–Eosin Staining
HMDB	Human Metabolomics Database
IMF	Intramuscular Fat
KEGG	Kyoto Encyclopedia of Genes and Genomes
KNN	k-Nearest Neighbor Algorithm
LC-MS	Liquid Chromatography–Mass Spectrometry Coupling
LDA	Linear Discriminant Analysis
LEfSe	Effect Size of Linear Discriminant Analysis
MS30	Experimental Group (mixed microsilage, replacing 30% whole-plant corn microsilage)
MS60	Experimental Group (mixed microsilage, replacing 60% whole-plant corn microsilage)
MS90	Experimental Group (mixed microsilage, replacing 90% whole-plant corn microsilage)
MUFA	Monounsaturated Fatty Acid
NDF	Neutral Detergent Fiber
NMDS	Non-metric Multidimensional Scaling Analysis
OPLS-DA	Orthogonal Partial Least Squares Discriminant Analysis
OTU	Operational Taxonomic Unit
OM	Organic Matter
PCA	Principal Component Analysis
PCoA	Principal Coordinate Analysis
PUFA	Polyunsaturated Fatty Acid
QC	Quality Control
SFA	Saturated Fatty Acid
TFAs	Total Fatty Acids
uNDF	Undigested Neutral Detergent Fiber
VIP	Variable Importance in Projection
